# Supplementary material for: Trends in Congenital Syphilis Incidence and Mortality in Brazil’s Southeast Region: A Time-Series Analysis (2008–2022)
Source: Epidemiologia (Basel). 2025 May 5;6(2):22. doi: 10.3390/epidemiologia6020022 (PMC12101142; doi:10.3390/epidemiologia6020022)
Supplement: Supplementary file 1 [file epidemiologia-06-00022-s001.zip › epidemiologia-3572684-supplementary.pdf]

Supplementary Material

Table S1. Number of live births in Brazil's Southeast Region and the states of Espírito Santo, Minas Gerais, Rio de Janeiro, and São Paulo, from 2008 to 2022.

Year	Espirito Santo	Minas Gerais	Rio De Janeiro	São Paulo	Total Southeast Region
2008	51,852	260,916	215,844	601,795	1,130,407
2009	51,457	252,676	216,625	598,473	1,119,231
2010	51,853	255,126	215,262	601,352	1,123,593
2011	53,053	259,863	220,603	610,222	1,143,741
2012	52,835	260,544	222,859	616,608	1,152,846
2013	54,065	258,635	224,031	610,896	1,147,627
2014	56,548	267,130	233,584	625,687	1,182,949
2015	56,941	268,305	236,960	634,026	1,196,232
2016	53,413	253,520	219,129	601,437	1,127,499
2017	55,846	260,959	223,224	611,803	1,151,832
2018	56,721	263,640	220,499	606,146	1,147,006
2019	54,925	256,892	207,989	583,191	1,102,997
2020	53,767	247,198	199,124	552,310	1,052,399
2021	52,493	242,136	189,866	525,239	1,009,734
2022	51,729	235,063	180,369	512,520	979,681

Table S2. Temporal trend of congenital syphilis incidence in children under one year of age in Brazil's Southeast Region and the states of Espírito Santo, Minas Gerais, Rio de Janeiro, and São Paulo, from 2008 to 2022.

	Year	APC (95% CI) *	*p*	AAPC (95% CI) **	*p*	Interpretation
Total Southeast Region						
	2008–2022			12.8 (11.0; 14.7)	≤0.001	Growing
	2008–2016	19.9 (17.4; 22.5)	≤0.001			Growing
	2016–2022	4.0 (0.7; 7.5)	0.022			Growing
Espirito Santo						
	2008–2022			14.0 (6.2; 22.4)	≤0.001	Growing
	2008–2016	27.1 (21.6; 32.8)	≤0.001			Growing
	2016–2020	−12.8 (−28.8; 6.7)	0.152			Stationary
	2020–2022	26.3 (−15.7; 89.4)	0.214			Stationary
Minas Gerais						
	2008–2022			21.4 (16.9; 26.1)	≤0.001	Growing
	2008–2017	33.4 (28.2; 38.8)	≤0.001			Growing
	2017–2022	2.5 (−6.9; 12.9)	0.581			Stationary
Rio de Janeiro						
	2008–2022			10.9 (7.8; 14.1)	≤0.001	Growing
	2008–2012	19.5 (8.6; 31.6)	0.001			Growing
	2012–2022	7.6 (5.1; 10.2)	≤0.001			Growing
São Paulo						
	2008–2022			14.1 (11.9; 16.3)	≤0.001	Growing
	2008–2015	24.5 (20.7; 28.4)	≤0.001			Growing
	2015–2022	4.6 (1.4; 7.9)	0.008			Growing

* APC, Annual Percent Change; ** AAPC, Average Annual Percent Change.

Table S3. Temporal trend of congenital syphilis mortality in children under one year of age in Brazil's Southeast Region and the states of Espírito Santo, Minas Gerais, Rio de Janeiro, and São Paulo, from 2008 to 2022.

	Year	APC (95% CI) *	*p*	AAPC (95% CI) **	*p*	Interpretation
Total Southeast Region						
	2008–2022			11.9 (1.3; 23.7)	0.026	Growing
	2008–2010	67.8 (−12.7; 222.9)	0.103			Stationary
	2010–2017	13.0 (1.2; 26.2)	0.034			Growing
	2017–2022	−5.9 (−18.7; 8.8)	0.352			Stationary
Espirito Santo ***						
Minas Gerais						
	2008–2022			21.9 (10.9; 33.9)	≤0.001	Growing
Rio de Janeiro						
	2008–2022			10.1 (2.6; 18.2)	0.006	Growing
	2008–2012	41.1 (11.1; 79.1)	0.009			Growing
	2012–2022	−0.2 (−5.9; 5.8)	0.938			Stationary
São Paulo						
	2008–2022			20.8 (5.7; 38.2)	0.005	Growing
	2008–2010	140.8 (−11.8; 557.8)	0.079			Stationary
	2010–2022	7.7 (1.5; 14.3)	0.018			Growing

* APC, Annual Percent Change; ** AAPC, Average Annual Percent Change. *** Temporal trend was not performed due to the absence of deaths in 2021 and 2022.
